# The Inflammasome in Reproductive Biology: A Promising Target for Novel Therapies

**DOI:** 10.3389/fendo.2020.00008

**Published:** 2020-01-28

**Authors:** Juan Pablo de Rivero Vaccari

**Affiliations:** ^1^Department of Neurological Surgery and The Miami Project to Cure Paralysis, University of Miami Miller School of Medicine, Miami, FL, United States; ^2^Center for Cognitive Neuroscience and Aging, University of Miami Miller School of Medicine, Miami, FL, United States; ^3^InflamaCORE, LLC, Miami, FL, United States

**Keywords:** inflammasome, fertility, inflammation, caspase-1, reproduction

## Abstract

The inflammasome is a key regulator of innate immunity involved in the inflammatory response to infections as well as disease through the activation of caspase-1 and the processing of the inflammatory cytokines interleukin (IL)-1β and IL-18. Even though the inflammasome was first described in the context of infections, most research in recent years has focused on targeting the inflammasome as a therapeutic option in sterile inflammatory events. Recent evidence indicates a clear involvement of the inflammasome in Reproductive Biology such as infertility and preeclampsia. In this mini-review, I summarize the current findings on the inflammasome that have been described in the field of Reproductive Biology and highlight the potential that the inflammasome has as a novel therapeutic option in this field. The topics covered in this review as it pertains to the inflammasome field cover the literature published on male and female infertility, endometriosis, preeclampsia, placental inflammation, and reproductive senescence.

## The Inflammasome

**Figure 1:**
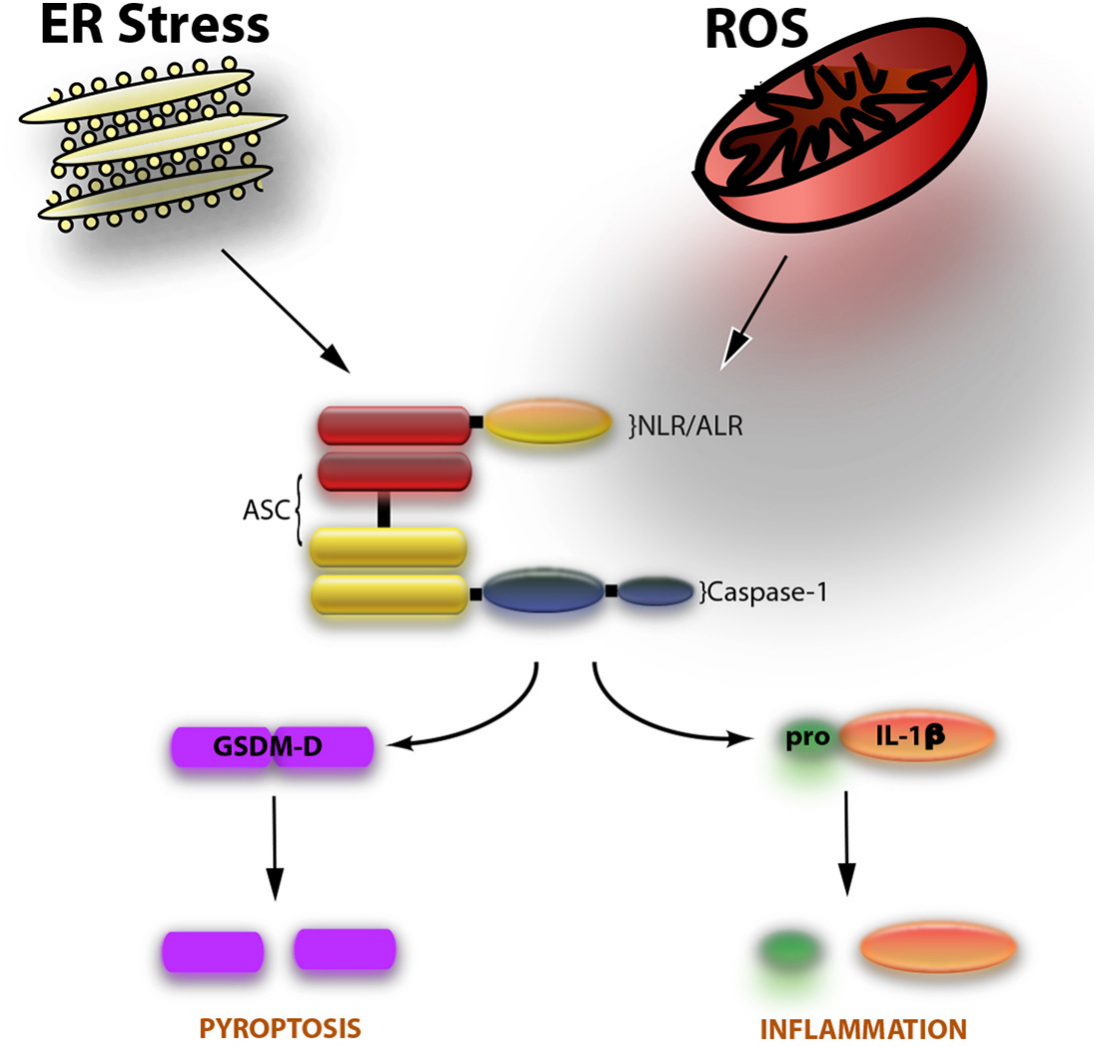
The inflammasome is comprised of caspase-1, ASC, and an NLR such as NLRP1 or NLRP3. Two events involved in the activation of the inflammasome are endoplasmic reticulum (ER) stress and the formation of reactive oxygen species (ROS). Upon activation of the inflammasome, caspase-1 is cleaved. Once cleaved (activated), caspase-1 goes on to cleave the pro-inflammatory cytokine IL-1β to induce inflammation. In addition, the substrate of pyroptosis (inflammasome-mediated cell death) gasdermin-D (GSDM-D) is cleaved. GSDM-D cleavage results in the formation of pores through which IL-1β is then released as well as cell death.

The inflammasome is a multiprotein complex with a dual role, one on inflammation and the other one on cell death. The most studied role of the inflammasome involves the activation of the cysteine aspartase caspase-1, resulting in the processing of the pro-inflammatory cytokines interleukin (IL)-1β and IL-18 (1). The most recently identified role of the inflammasome is the cell death mechanism of pyroptosis, which involves the cleavage of gasdermin-D and the release, but not activation, of IL-1β (2). The inflammasome is comprised of three basic components: a nucleotide oligomerization domain (NOD)-like receptor (NLR) such as NLRP1, NLRP2, or NLRP3 as well as the adaptor protein known as apoptosis-associated speck-like protein containing a caspase activating recruitment domain (ASC) and the inflammatory cysteine protease caspase-1 (Figure 1).

## Initial Steps in the Field of Inflammasome Research

The inflammasome was initially discovered by the late Tschopp and colleagues in 2002 as a multiprotein complex involved in the activation of caspase-1, which is responsible for activating IL-1β and IL-18 (1). Most of the initial studies on the inflammasome started focusing on bacterial infections (3). Then these studies were further extended to the role of inflammasomes in viral (4) and fungal infections (5, 6) as well as autoimmune diseases (7). In the mid 2000s, the first studies on the inflammasome in a sterile event were carried on vitiligo (8) and central nervous system injury (9). Since then, the inflammasome field has started to expand into other indications such as atherosclerosis (10), diabetes (11), nephropathies (12), liver diseases (13), aging (14, 15) as well as in the field of reproductive biology (16, 17), which extent even to the effects of obesity and the inflammatory contribution of the inflammasome to male subfertility (18).

## The Inflammasome in Reproductive Biology

In the context of reproductive biology, the inflammasome has been studied in areas as diverse as female (19) and male infertility (16, 17), fetal growth (20), endometriosis (21), preeclampsia (22), gestational diabetes (23), perinatal depression (24), placental inflammation (25), preterm births (26), and reproductive senescence (27) (Table 1).

**Table 1:** Conditions associated with inflammasome activation in the field of Reproductive Biology.

**Condition**	**Findings**	**References**
Female infertility	NLRP3 gene polymorphism associated with female infertility	(19)
Male infertility	Inflammasome inhibition improves sperm motility in spinal cord injured men	(16, 17)
Endometriosis	Inflammasome signaling proteins are elevated in the endometrium of females with recurrent pregnancy loss	(28)
Preeclampsia	The NLRP3 inflammasome contributes to the inflammatory response seen in preeclampsia	(25, 29, 30)
Preterm births	Caspase-1, ASC, and IL-1β genes are elevated in preterm birth mice	(26)
Reproductive senescence	Inflammasome proteins are carried in EV released by female reproductive organs that reach the brain, contributing to brain inflammation	(27)

### Infertility

Effective fertility requires a fine balance between pro- and anti-inflammatory mediators. Thus, an imbalance in the inflammatory response during fertilization and early embryogenesis dooms the process toward pregnancy failure (31). Witkin and colleagues showed that a polymorphism in the gene encoding for NLRP3 (*CIAS1*) is associated with female infertility. Interestingly, this polymorphism increased the likelihood of mycoplasma infection-associated female infertility (19). Moreover, another role for NLRP2 was also described for infertility. The NLRP2 inflammasome was first described to be formed in the nervous system (32). In the context of fertility, NLRP2 regulates oocyte quality, which is involved in age-associated fertility loss (33). In addition, a role for NLRP3 in the immune response in the testes has also been described (34). In addition, in the sperm of patients with spinal cord injury, inflammasome proteins are elevated (16), and this increase in inflammasome protein expression is consistent with decrease sperm motility that is improved by inhibition of ASC (16). In a rodent model of spinal cord injury, similar findings have been recently reported (35).

### Endometriosis

An abnormal imbalance between pro- and anti-inflammatory proteins in the endometrium results in recurrent miscarriages. Inflammatory proteins like tumor necrosis factor, IL-6, IL-10, and interferon-γ are dysregulated in women with recurrent pregnancy loss (36). Thus, highlighting the importance of an adequate pro- to anti-inflammatory milieu. Similarly, significant research has started to be published in the rea of the inflammasome and the endometrium (37). Accordingly, NLRP3, caspase-1, ASC, IL-1β, and IL-18 are increased in the endometrium of women with recurrent pregnancy loss (28). Thus, future therapeutic alternatives that aim to rebalance the pro- to anti-inflammatory milieu in the endometrium should also consider the inflammasome as part of the equation.

### Preeclampsia and Placental Inflammation

A disorder associated with hypertension and proteinuria starting on the 20th week of pregnancy (38), preeclampsia has a significantly heightened inflammatory response in which the inflammasome plays a contributing role (39). In regards to inflammasome regulation in preeclampsia, Weel and colleagues showed that the NLRP3 inflammasome is upregulated, and that it contributes to the damaging effects of inflammation present in preeclampsia (29), a finding that was then corroborated by Stodle et al. who showed that cholesterol and uric acid crystals activated the NLRP3 inflammasome in preeclampsia (30). A similar role for NLRP3 was suggested in a model of nanosilica-induced placental inflammation in rodents, but not for ASC (25). However, ASC is significantly increased in the amniotic fluid of women who undergo spontaneous labor at term (40). More recently, extracellular vesicles (EV) have been shown to activate the inflammasome in trophoblasts, thus promoting preeclampsia (41). Moreover, in women with anti-phospholipid syndrome, NLRP3 and ASC are responsible for placental dysfunction that increases adverse pregnancy outcomes (42). For instance, ASC specks have been detected in choriodecidual leukocytes isolated from women who underwent spontaneous labor at term (43).

In addition, exacerbated inflammation in the placenta is associated with fetal growth restriction (44), and protein levels of caspase-1 and IL-1β were elevated in cytotrophoblasts exposed to uric acid crystals, suggesting that inflammasome activation may contribute to placental inflammation by exposure to uric acid crystals, which are known to be associated with fetal growth restriction, preeclampsia and inflammasome activation. Taken together, these findings indicate a clear role for the inflammasome in preeclampsia and placental inflammation.

### Reproductive Senescence

Reproductive senescence in females is characterized by heightened inflammation, which makes females more prone to the development of certain diseases. Inflammasome proteins have been shown to be present in EV (45). Interestingly, in reproductive senescent females, EV containing a cargo of inflammasome proteins originate in the female reproductive organs such as the ovaries; EV are then transported through the bloodstream to the nervous system by crossing the blood brain barrier, resulting in inflammasome activation in the brain (27). This heightened inflammasome activation in the brain makes females more susceptible to the damaging effects of central nervous system events such as stroke.

## Therapeutic Potential of the Inflammasome

As a result of inflammasome involvement in several indications affecting several organ systems, the inflammasome is well-poised for the development of therapeutic interventions that can improve outcomes in a variety of diseases. Recently, as a result of this tremendous therapeutic potential, Big Pharma and the Biotechnology Industry have garnered special interest in licensing and developing therapeutic interventions that are meant to inhibit the inflammasome in a variety of diseases such as neurodegenerative diseases, liver diseases or gout, among others. The therapeutic potential of the inflammasome is so vast that it has been proven difficult to decide what indication to choose for clinical trials targeting the inflammasome.

Testing therapeutic interventions aimed at inhibiting inflammasome activation is of utmost importance since the ultimate role should be to gain a better mechanistic understanding so that efficient and more specific therapies can be eventually tested in patients. In the field of Reproductive Biology, miR-520c-3p has been shown to inhibit the NLRP3 inflammasome in preeclampsia (46). In addition, the NLRP3 inhibitor MCC950 has been shown to reduce preterm birth by 35.7% and neonatal mortality by 26.7% (47). Similarly, the NLRP3 inflammasome inhibitor glibenclamide also decreases inflammasome activation in human trophoblasts, thus highlighting the therapeutic potential of the NLRP3 inflammasome for the treatment of placental disorders (22).

Moreover, other inflammasomes such as the NLRP1 and AIM2 inflammasomes are also promising targets in this field. For instance, omega-3 fatty acids inhibit NLRP1 and AIM2 inflammasome activation and trophoblast cathepsin S release into the cytosol from lysosomes, thus reducing preterm birth associated with infection and inflammation (48).

Taken together, these findings in the area of Reproductive Biology highlight the important role of the inflammasome, and indicate that therapeutic targeting of the inflammasome is a viable option to treat reproduction-related problems. Current evidence points at NLRP1, NRLP2, NLRP3, AIM2, caspase-1, ASC, and IL-1β as potential targets for therapeutic intervention in this field.

## Future Directions and Conclusions

Inflammasome research in the field of Reproductive Biology needs to focus on more mechanistic insights beyond understanding the expression of inflammasome signaling proteins like caspase-1, ASC, and IL-1β (Figure 1). Future research should take a deeper look into the potential mechanisms of inflammasome activation such as extracellular potassium levels (22); the role of oxidative stress on inflammasome activation (49, 50); or whether the inflammasome-mediated process of pyroptosis, or the non-canonical inflammasome pathways, involving caspase-11 in rodents (caspase-4/5 in humans), or caspase-8 are involved in conditions associated with reproduction. To this extent, a recent article has been published showing that hypoxia and endoplasmic reticulum stress activate the NLRP3 inflammasome in primary human trophoblasts, resulting in increased expression of Thioredoxin-interacting protein (TXNIP), a key regulator of inflammasome activation (51). Moreover, these findings were consistent with increased cleavage of caspase-1 and GSDM-D, thus indicating that placental pyroptosis contributes to the systemic release of factors involved in preeclampsia (52).

In conclusion, whether it involves female or male reproductive biology, the data published so far indicate that it is critical to maintain an adequate ratio of pro-inflammatory to anti-inflammatory proteins to increase the possibility of successful reproduction. Thus, targeting the inflammasome to decrease the pro-inflammatory environment is a promising approach, but further research in the area of biomarkers will be useful in gaining a better understanding as to what are the right protein concentrations for relevant pro-inflammatory and anti-inflammatory markers that can be used to help patients with reproductive problems. For instance, one of such studies has been carried looking at increased ASC levels in amniotic fluid obtained from women with clinical chorioamnionitis at term (53). Therefore, further research should focus on mechanistic insights with the goal of developing better therapies and on biomarkers with the goal of diagnosis and monitoring patients once those treatments are tested in clinical trials or delivered to patients in the clinical setting.

## Author Contributions

JR contributed fully to the writing of this article.

### Conflict of Interest

JR is a co-founder and managing member of InflamaCORE, LLC and has licensed patents on inflammasome proteins as biomarkers of injury and disease as well as on targeting inflammasome proteins for therapeutic purposes. JR is a Scientific Advisory Board Member for ZyVersa Therapeutics.
